# Supplementary material for: Rapid turnover of life-cycle-related genes in the brown algae
Source: Genome Biol. 2019 Feb 14;20:35. doi: 10.1186/s13059-019-1630-6 (PMC6374913; doi:10.1186/s13059-019-1630-6)
Supplement: Supplementary file 3 — Protein alignments, corresponding Gblocks html file and CDS sequences for the lineage conserved gametophyte-biased genes. (ZIP 2109 kb) [file 13059_2019_1630_MOESM3_ESM.zip › Con_GA_alignments/Ec-01_001140.aa_ali.fasta-gb.htm]

Ec-01\_001140.aa.fasta.trans.aa\_ali.fasta


## Gblocks 0.91b Results

Processed file: **Ec-01\_001140.aa.fasta.trans.aa\_ali.fasta**  
Number of sequences: **2**  
Alignment assumed to be: **Protein**  
New number of positions: **396** (selected positions are underlined in blue)

```
                         10        20        30        40        50        60
                 =========+=========+=========+=========+=========+=========+
Ec-01_001140     MVTRGRCEKTRIDFYRSSASATLLQLQQYLRNAVGCHLPVSRYEEHGRLYFYNLDTGETT
MSTRG.49241.1    ------------------------------------------------------------
                                                                             


                         70        80        90       100       110       120
                 =========+=========+=========+=========+=========+=========+
Ec-01_001140     WDPPPQSREGETSGPPPAATNRSGAVTVRSRSSVILSKGGKIQFGRSCSAVSSRSEAPKH
MSTRG.49241.1    ------------------------------------------------------------
                                                                             


                        130       140       150       160       170       180
                 =========+=========+=========+=========+=========+=========+
Ec-01_001140     PTVVHKGGRLGTTDELDDGGGRTLKTGTTEGAVVRSMLHRASTLPARNVEGESPGAEREG
MSTRG.49241.1    ------------------------------------------------------------
                                                                             


                        190       200       210       220       230       240
                 =========+=========+=========+=========+=========+=========+
Ec-01_001140     YTSSRQAPGKKDGDETKHVLGSPQRSVGCPEESTPLPKWSGLKKPTRQRAKHVEEPPTSK
MSTRG.49241.1    -------------------------EACCP------------------RTQRSKIPLKSE
                                                                 ############


                        250       260       270       280       290       300
                 =========+=========+=========+=========+=========+=========+
Ec-01_001140     PAPSSQGNHLVPSSLQQAEQWLATNEPESELGRP-YRRRGPDQTPPKPDFSLGTRETSGR
MSTRG.49241.1    GPESSSGATRVATSLLRAQNWLAQNEPEVELERPRSRTLTPDRTRPRPKSSRGATRLS--
                 ##################################  ######################  


                        310       320       330       340       350       360
                 =========+=========+=========+=========+=========+=========+
Ec-01_001140     FATPTHVEDASQKEGVPMHEPPAATTARLRGQAAAVREAMELRRKEAEEMTGRPEITRRG
MSTRG.49241.1    -CKPVGSSDE-RVMACRAEGSGATTTARLRGRAAAVWASVQAQRREEEEMTGKPEITRLG
                                       ######################################


                        370       380       390       400       410       420
                 =========+=========+=========+=========+=========+=========+
Ec-01_001140     RNKQRSVDDLLLFQRNAEASRRRRHDERETHENKLVTGRPETCRRSAVLVKRMRRGGGND
MSTRG.49241.1    RSKRRSVDDLFLFQRNVEDSRRRKRDQREAHEKKLVTGRPETCGRSTALVKQMRRDGGAD
                 ############################################################


                        430       440       450       460       470       480
                 =========+=========+=========+=========+=========+=========+
Ec-01_001140     -AHLPTSQRLHSDAGEQRRRRDTQQARVETAIRRRANPNISTRSKSLPRER--GDVARRL
MSTRG.49241.1    RTQLSTSKRLYGDAREQRRRQETLQMQVDTAIRRRANPTLSVRSRSLPRDRSRGDAARRL
                    ################################################  #######


                        490       500       510       520       530       540
                 =========+=========+=========+=========+=========+=========+
Ec-01_001140     YEQAILSREGAHYREQLSRK-PPRGHTFRPEIDQRSALLAQRRRDRWAS-VGLDKRGR--
MSTRG.49241.1    YEQAIASRDDALQRERYAAQNLPRGVTFHPNIHQRSTFLAQRRRERWASAVGLGVPGIET
                 ###############       ###########################           


                        550       560       570       580       590       600
                 =========+=========+=========+=========+=========+=========+
Ec-01_001140     -------RGGTAVQEFLLAEGTLYDRRRQERQSIQEELALRRPNPTVNRHSRRLLRAAGK
MSTRG.49241.1    GSVATADPAGAAVQEFLLAEGALYDRRRRERQLRQEELALRSKIPAVNRHSRRLLQKAER
                          ###################################################


                        610       620       630       640       650       660
                 =========+=========+=========+=========+=========+=========+
Ec-01_001140     TGRSPGTQSEE-TRSRASEAVEAEQYEFSPRLEGLGTSQKMLESRYGRDAIPSMQQRAKD
MSTRG.49241.1    MGRSLGVQDDSRVPEDVADVDSAAGYDFSPRLEALDTSKRMLESRYGRGSIPSMEQRTKE
                 ########              ######################################


                        670       680       690       700       710       720
                 =========+=========+=========+=========+=========+=========+
Ec-01_001140     REVEILERRRSQSVEQDKEDRSFVARRASRRSSASESPEHGQD--EPRSAKSFSRKS---
MSTRG.49241.1    RERERLEK-EAQLDERNKENRDFVARRVARSCPENGDAEHFRGAGIYERTKEYSRQKETR
                 #######    #############################                    


                        730       740       750       760       770       780
                 =========+=========+=========+=========+=========+=========+
Ec-01_001140     WSAAR------------LSPSCRRE-----------------------------------
MSTRG.49241.1    WASEREALLAEELADCTFSPEFRTKRRGRSRYRQQASGVNGPNSSLAGEVSDVVADEEVV
                                                                             


                        790       800       810       820       830       840
                 =========+=========+=========+=========+=========+=========+
Ec-01_001140     ----------------------SGGGQDT-------------------------------
MSTRG.49241.1    PKVGTVEMDGSASRSLDSSGCNGGGSRRSGPLRDGEGSSGLPTRRRRRPKQQTTIEDEGS
                                                                             


                        850       860       870       880       890       900
                 =========+=========+=========+=========+=========+=========+
Ec-01_001140     ------------------------------------------------------------
MSTRG.49241.1    GVGLFPFFGEEDVVEDAGGSGRSGDYSAGSAGGRGPRKDSDRARGRADEETRSLSALLSR
                                                                             


                        910       920       930       940       950       960
                 =========+=========+=========+=========+=========+=========+
Ec-01_001140     ------------------------------------------------------------
MSTRG.49241.1    RPSGGAGGDGYSSTQDDYSVYDSTEDGSDHFLLETGDTIAATLSASARNDGSRRSSTSGR
                                                                             


                        970       980       990      1000      1010
                 =========+=========+=========+=========+=========+==
Ec-01_001140     ----------------------------------------------GRPPKP
MSTRG.49241.1    AVTAVTGEGTPIDDDSAASPLPSGWTAFTSPEGWAYFFNEETSAVQWKRPTR
```

```
Parameters used
Minimum Number Of Sequences For A Conserved Position: 2
Minimum Number Of Sequences For A Flanking Position: 2
Maximum Number Of Contiguous Nonconserved Positions: 8
Minimum Length Of A Block: 10
Allowed Gap Positions: None
Use Similarity Matrices: Yes
```

```
Flank positions of the 9 selected block(s)
Flanks: [229  274]  [277  298]  [323  420]  [424  471]  [474  495]  [503  529]  [550  608]  [623  667]  [672  700]  

New number of positions in Ec-01_001140.aa.fasta.trans.aa_ali.fasta-gb:  396  (39% of the original 1012 positions)
```
